# Easing the burden: a pilot study on the impact of mindfulness on the mental health of Brazilian medical students

**DOI:** 10.1186/s12909-025-07726-2

**Published:** 2025-08-01

**Authors:** Vinicius Vieira Neves, Daniel Teixeira dos Santos, Marcelo Demarzo, Lia Antico, Cynthia de Almeida Brandão Meirelles, Ana Rosa Airão Barboza

**Affiliations:** 1 Universidade do Grande Rio Professor José de Souza Herdy, Rio de Janeiro, Brazil; 2 University College London, London, UK; 3 Mindfulness Center at Brown University, Providence, RI USA; 4 Department of Neurology, Hospital de Clínicas de Porto Alegre, Porto Alegre, Rio Grande do Sul Brazil; 5 Department of Neurology, Santa Casa de Misericórdia de Porto Alegre, Porto Alegre, Rio Grande do Sul Brazil; 6 Department of Preventive Medicine, Graduate Program in Collective Health, Universidade Federal de São Paulo, São Paulo, SP Brazil; 7 Mente Aberta - Brazilian Center for Mindfulness and Health Promotion, Departamento de Medicina Preventiva, Universidade Federal de São Paulo, São Paulo, SP Brazil; 8 Department of Behavioral and Social Sciences, Brown University, Providence, RI USA; 9 Department of Pediatric Neurology, Universidade Federal do Estado do Rio de Janeiro, Rio de Janeiro, Brazil

**Keywords:** Mindfulness, Students, medical, Depression, Anxiety, Perceived stress, Mental health, Pilot projects, Intervention studies, Mindfulness-based interventions, Education, medical

## Abstract

**Background:**

Mental health disorders, such as anxiety and depression, are more prevalent in medical students than in the general population. Mindfulness-based interventions (MBIs) have shown evidence of effectiveness in treating these conditions. However, findings among medical students are mixed, particularly in Brazilian samples. This study aims to evaluate the feasibility and preliminary effects of the Mindfulness-Based Health Promotion (MBHP) program on perceived stress, mindfulness, and symptoms of anxiety and depression in Brazilian medical students.

**Methods:**

This single-arm pilot clinical trial involved medical students participating in the MBHP program for 2.5 h per week over eight weeks. A convenience sample from a university’s interest group was recruited through referrals. Those aged ≥ 18, enrolled in medical school, and owning a smartphone were eligible. Outcomes were assessed pre- and post-intervention. Feasibility was evaluated through recruitment and retention rates. Depressive and anxiety symptoms were measured using the Patient Health Questionnaire-9 (PHQ-9) and the Generalized Anxiety Disorder-7 (GAD-7); perceived stress and mindfulness were assessed using the Perceived Stress Scale-10 (PSS-10) and the Five Facet Mindfulness Questionnaire (FFMQ). Data were analyzed using descriptive statistics and the Wilcoxon Signed-Rank test.

**Results:**

All 13 eligible participants enrolled and attended at least 50% of the sessions, resulting in recruitment and retention rates of 100%. Participants (76.9% female, 92.3% Caucasian, mean age = 23.6 years) showed significant reductions in depressive (*p* =.001; *r* =.62) and anxiety (*p* =.014; *r* =.41) symptoms. Overall mindfulness increased significantly (*p* =.001; *r* =.62), as did four of its five facets: Observe (*p* =.012; *r* =.49), Act with Awareness (*p* =.009; *r* =.51), Non-judgement (*p* =.046; *r* =.39) and Non-reaction (*p* =.007; *r* =.52). Perceived stress was not significantly reduced (*p* =.059; *r* =.37).

**Conclusion:**

MBHP was feasible and may reduce anxiety and depression while enhancing mindfulness in Brazilian medical students. Higher-quality randomized trials with larger samples and longer follow-up are needed to confirm these preliminary findings.

**Registration:**

The trial was retrospectively registered with the *Registro Brasileiro de Ensaios Clínicos* (ReBEC) on February 26, 2025, under registration number RBR-44cvfnq.

## Background

Due to the inherent demands of healthcare training, medical school presents a particularly challenging environment, predisposing students to mental health issues. For instance, a meta-analysis of 167 cross-sectional (*n* = 116,628) and 16 longitudinal (*n* = 5,728) studies from 43 countries found a 27.2% prevalence of depressive symptoms and an 11.1% prevalence of suicidal ideation among medical students [1]. Anxiety appears to be even more prevalent, as evidenced by a meta-analysis of 69 cross-sectional studies including 40,348 medical students, which reported a global anxiety prevalence of 33.8% [2]. Comparatively, medical students generally exhibit higher levels of depression and anxiety, as well as poorer overall mental health, than age-matched nonmedical students [3–5].

Data on Brazilian medical students is relatively scarce; however, existing studies indicate a similar trend [6–8]. A 2017 meta-analysis of 57 cross-sectional studies (*n* = 18,015) reported prevalence rates of depression and anxiety at 30.6% and 32.9%, respectively, while elevated perceived stress was observed in 49.9% of participants, based on self-reported measures of perceived stress [7]. Additionally, a study conducted at the same university as the present study found an association between depressive symptoms and alcohol dependence [9]. In line with this, a recent cross-sectional study reported that 36.48% of students had used the school’s mental health services, and 85.13% sought emotional support from the institution and friends [8]. A qualitative analysis of their findings revealed that anxiety, depression, and stress were among the primary reasons for seeking the school’s mental health services.

Some authors suggest that cultural factors, such as the belief that medical schools must be demanding to push students perceived as ‘weak’ to drop out, may contribute to this issue [10, 11]. Other proposed factors include the competitiveness of medical education, perfectionism, the increasing volume of learning materials, and exposure to severely ill and dying patients [11, 12]. The feelings of inadequacy that arise in this high-pressure environment, where mistakes can have serious consequences, often lead to heightened self-criticism, which may culminate in depressive and anxiety symptoms or even suicidal ideation [13].

Additionally, mental health issues during medical school can have long-term consequences that extend beyond the individual and the period in which they arise. For instance, perceived stress in medical school has been identified as an independent predictor of post-graduation mental health issues requiring treatment [14]. One such issue is physician burnout, which the World Health Organization (WHO) defines as “a syndrome resulting from chronic workplace stress that has not been managed successfully” [15]. Other sources describe burnout as “a psychological syndrome characterized by a negative emotional reaction to one’s job as a consequence of prolonged exposure to a stressful work environment,” emphasizing the role of the work environment as a major contributing factor [16, 17]. Burnout is significantly associated with poorer patient care, increased medical errors [18], reduced patient safety, and a higher risk of malpractice claims [19, 20]. It also contributes to reduced clinical hours, high physician turnover, and is estimated to cost the United States $4.6 billion annually [21].

Although these concerns date back to 1936 [22], the COVID-19 pandemic substantially exacerbated already high burnout levels among healthcare professionals [23], and advances in treatment interventions have only emerged more recently [11, 24]. To avoid placing the burden solely on one aspect of the issue, these interventions target two main areas: the individual and the educational environment, both of which have shown promising results [24–26]. Interventions focusing on the educational environment primarily involve changes to the medical school curriculum structure [24], while those targeting the individual level aim to enhance medical students’ coping skills [27]. Examples include relaxation training, Mindfulness-Based Stress Reduction (MBSR), self-hypnosis, discussion and educational groups on self-care, and support groups [25, 26, 28–31]. Among these approaches, mindfulness-based interventions (MBIs) show the greatest promise in both mental health and medical education [32–35].

Mindfulness is often defined as the awareness that arises from intentionally and nonjudgmentally paying attention to present-moment experience [36]. To standardize training for cultivating this awareness, Jon Kabat-Zinn developed Mindfulness-Based Stress Reduction (MBSR) in 1979. MBSR is an eight-week, group-based intervention led by certified instructors trained in mindfulness-based skills [36]. Since then, several adaptations of MBSR have been developed for different populations and settings, including Mindfulness-Based Cognitive Therapy (MBCT), Mindfulness-Based Eating Awareness Training (MB-EAT), Mindfulness-Based Relapse Prevention (MBRP), Mindfulness-Based Blood Pressure Reduction (MB-BP), and Mindfulness-Based Health Promotion (MBHP), the latter of which was employed in the present study [37–43].

These programs have demonstrated improved outcomes in clinical research. An individual patient data meta-analysis of nine randomized controlled trials (RCTs) (*n* = 1,258) found that the MBCT program was noninferior to pharmacologic treatment in preventing depression relapse [44]. This study influenced the National Institute for Health and Care Excellence (NICE) guidelines for adult depression management in the United Kingdom, leading to a formal recommendation of MBCT for individuals with three or more previous depressive episodes [45]. Regarding anxiety disorders, a recent RCT (*n* = 276) comparing MBSR with escitalopram, a first-line pharmacologic treatment, found MBSR to be noninferior to the medication [46].

The MBHP program has also been evaluated in multiple randomized controlled trials and feasibility studies involving diverse populations, such as older adults [47], healthcare workers [48], teachers [49, 50], police officers [51], caregivers [52], and college students [53]. Published evidence consistently demonstrates improvements in mindfulness, stress reduction, depression, anxiety, and quality of life [54–56].

Specifically among medical students, although data remains somewhat limited, findings follow a similar trend. A few reviews and meta-analyses have reported reductions in depression, anxiety, and stress, along with increases in mindfulness [32–34]. However, significant heterogeneity exists between studies, the risk of bias—particularly selection bias—is high, the quality of evidence is low to moderate, and most studies have been conducted in the United States (US) [32–34]. As of 2014, mindfulness-related wellness activities were offered in approximately two-thirds of US medical schools [35]. It is worth noting, however, that many of these were informal wellness offerings, small-scale programs, or pilot projects, and only available to a limited subset of students.

A few studies have examined MBIs in Brazilian medical students, with some showing no effects on mental health outcomes, while others have demonstrated promising results [57–59]. Additionally, the MBHP program, which focuses specifically on health promotion and quality of life, has only been tested in healthcare workers in Colombia [48, 60]. Therefore, this study aims to contribute to the literature of MBIs among Brazilian medical students by assessing the feasibility and preliminary effects of the MBHP program on their mental health, particularly in relation to depressive symptoms, anxiety symptoms, perceived stress, and mindfulness.

## Methods

### Study design and sample

This was a non-controlled, single-arm pilot clinical trial assessing mental health variables before and after the MBHP program in Brazilian medical students. The trial was retrospectively registered with the *Registro Brasileiro de Ensaios Clínicos* (ReBEC) (approved on February 26, 2025) under registration number RBR-44cvfnq. Primary outcomes included feasibility, depressive symptoms, anxiety symptoms, perceived stress, and mindfulness. Secondary outcomes consisted of five mindfulness subdimensions, described in detail below (see: *Description of measures*).

Participants were recruited in August 2018 through referrals from the university’s Integrative Medicine and Spirituality Interest Group. The intervention and assessments were conducted during September and October 2018, within the regular academic semester. Sessions were held once a week in the late evening, after regular medical school classes, to accommodate students’ academic schedules.

As hypothesis testing is not appropriate in feasibility pilot studies, no formal sample size calculation or power analysis was conducted, in accordance with established methodological guidance [61–63]. Sample sizes in pilot studies are typically based on practical considerations [64], as was the case in our study, which used a convenience sample of medical students who were members of the Integrative Medicine and Spirituality interest group. Rather than assessing efficacy, pilot studies aim to evaluate feasibility, acceptability, and conduct preliminary exploratory analyses to generate effect size estimates for adequately powering future trials [63].

Inclusion criteria were: (1) enrollment as a medical student at *Universidade do Grande Rio Professor José de Souza Herdy* (UNIGRANRIO); (2) ownership of a smartphone with WhatsApp^®^ and internet access; (3) age 18 years or older; and (4) provision of written informed consent.

Exclusion criteria were: (1) attending less than 50% of the program’s sessions and (2) worsening of a pre-existing psychiatric condition. Attendance at four or more sessions was used as the minimum for per-protocol analysis, consistent with standards in 8-session mindfulness interventions. This threshold would not apply to interventions of different lengths [65].

Although not pre-specified as an exclusion criteria, a safety screening procedure was implemented following best-practice recommendations [66] to minimize the risk of meditation-related adverse experiences. Prior to enrollment, all participants completed a clinical screening form assessing mental health history, current psychiatric medication use, and whether they were receiving care from a mental health counselor, psychiatrist or psychologist. This form was reviewed by the study psychiatrist, and participants whose responses raised potential safety concerns were invited for a clinical interview to assess their suitability for participation.

The study protocol was approved by the institutional review board at UNIGRANRIO under *Certificado de Apresentação para Apreciação Ética* (CAAE) number 86916318.5.0000.5283, on June 28, 2018. All procedures adhered to the ethical principles outlined in the Declaration of Helsinki.

### Intervention descriptions

Inspired by the MBSR, MBCT, and MBRP programs, the *Mente Aberta* Center at the *Universidade Federal do Estado de São Paulo* (UNIFESP), in partnership with Zaragoza University, developed the MBHP program in 2009. Currently, the center oversees instructor training and certification, and since 2014, over 700 instructors have been trained, and thousands of participants have completed the program in Brazil and Latin America. It was formally recognized by the European Association for Mindfulness-Based Approaches (eAMBA) in 2024. This eight-week program included weekly two-hour sessions conducted on the UNIGRANRIO campus. The program was led by two instructors certified to teach the MBHP program by the *Mente Aberta* Center.

Designed for the Hispanic/Latin American context, MBHP’s structure retains core practices from MBSR but introduces distinctive features to enhance accessibility and safety: it does not include a full-day silent retreat, replacing it with a structured two-hour silent practice session (Session 6). Sessions are shorter (1.5–2 h) and can be delivered in person or online to accommodate diverse schedules and contexts. Another key distinction is the inclusion of formal compassion and self-compassion practices in sessions 7 and 8, which aim to strengthen emotional regulation and cultivate a supportive attitude toward oneself and others. The program also explicitly integrates trauma-sensitive mindfulness principles, including pre-participation screening, trauma-informed group agreements, progressive exposure to practices, and clear referral pathways for participants requiring additional support. Examples of meditative practices include awareness of breathing, sounds or physical sensations, walking meditation, body scanning, mindful movement, and loving-kindness [67]. These practices were guided in class by the instructors, and recorded audio versions (15–20 min in length) were provided to participants for home practice, with recommendations to practice approximately once daily for six days a week.

Cultural adaptation is central to MBHP’s design, incorporating accessible, non-technical language, culturally relevant metaphors, and an emphasis on community orientation and relational well-being. This culturally responsive approach, combined with the strong scientific foundation and international endorsement, underscores MBHP’s suitability as an evidence-based, trauma-sensitive intervention aligned with high standards of safety, integrity, and effectiveness.

Sessions were co-facilitated by the instructors, who jointly led the mindfulness practices, inquiry, discussions and activities. Weekly session topics and core activities were as follows:

1. What is Mindfulness? Escaping autopilot: Mindfulness and autopilot are explored and defined experientially through the “mindful eating a raisin” practice. Body awareness practices, such as the body scan, are also introduced.
2. Awareness of the breath: Focused attention is deepened by introducing the breath as an anchor. The session also includes a discussion on the difference between primary and secondary suffering.
3. Mindfulness in daily life: Participants are introduced to mindful walking and explore how to bring mindfulness into routine activities, such as brushing their teeth.
4. Mindfulness for difficult situations: This session focuses on acceptance and the distinction between automatic reactions and skillful responses to stress during difficult or unwanted situations.
5. Mindfulness of the mind and thoughts: Participants enhance their awareness of thoughts as transient mental events and practice observing them without becoming entangled. The “train of thoughts” metaphor is introduced.
6. Silence: A structured two-hour silent practice session including several guided meditations and mindful walking, allowing participants to deepen experiential learning.
7. Mindfulness and compassion: Formal loving-kindness and self-compassion practices are introduced to foster a more caring and forgiving attitude toward oneself and others.
8. Mindfulness for life: The final session invites participants to reflect on their personal journey through the program and discuss strategies for maintaining mindfulness in daily life.

Additional teaching strategies included videos, metaphors, group activities, mindful eating, breathing exercises, guided reflections, and journaling. For example, the “train of thoughts” metaphor was incorporated to explain a key mindfulness principle in a tangible and accessible way. Group activities, held once or twice per session—such as mindful listening in small groups and mindfulness enactments (e.g., saying “Hello thought, thank you thought, goodbye thought”)—served a similar purpose while also fostering experiential learning. Lastly, participants were invited to complete reflective journaling at home on a weekly basis, using prompts such as “What did you notice during your practice this week?”, to support the integration of mindfulness into daily life.

### Description of measures

Data were collected in person one hour before the first MBHP session (baseline) and one hour before the final MBHP session (post-intervention) using printed questionnaires. We chose to conduct the final assessment before, rather than after, the last session to minimize disruption for the medical students and to mirror the procedure used in the first session, thereby reducing potential bias from completing the questionnaires after having meditated and participated in the session. Demographic variables included age, employment status, living arrangement, marital status, gender, medical school year, ethnicity, physical activity, use of botanical supplements, engagement in yoga or other stress management practices, and psychiatric medication use. At baseline, participants reported which psychiatric medications they were taking, and at post-program, they indicated whether they had changed the dose of, discontinued, or initiated any continuous psychiatric medications during the intervention.

Feasibility of the intervention was assessed through the percentage of eligible participants who enrolled (recruitment rate) and the percentage of participants who completed the program (retention rate).

Depressive symptoms were assessed using the Portuguese version of the Patient Health Questionnaire-9 (PHQ-9). This self-report instrument evaluates the presence of depressive symptoms over the past two weeks through nine Likert-scale items based on the Diagnostic and Statistical Manual of Mental Disorders IV (DSM-IV) criteria. The PHQ-9 has demonstrated good internal consistency (Cronbach’s α = 0,89) [68] and was validated in Brazil in 2008 [69]. Evidence suggests that scores ≥ 10 have high accuracy for diagnosing major depressive disorder [68, 70].

Anxiety symptoms were assessed using the Portuguese version of the Generalized Anxiety Disorder-7 (GAD-7) self-report questionnaire. Based on DSM-IV criteria, it evaluates the presence of generalized anxiety disorder symptoms over the past two weeks through seven Likert-scale items. The GAD-7 has demonstrated excellent internal consistency (Cronbach’s α = 0,92) and high accuracy for diagnosing the disorder when scores are ≥ 10 [71]. The Portuguese version has also demonstrated excellent internal consistency (Cronbach’s α = 0,916) [72].

Perceived stress was assessed using the Portuguese version of the Perceived Stress Scale-10 (PSS-10), a self-report questionnaire consisting of 10 Likert-scale items that measure the extent to which certain situations were perceived as stressful in one’s life over the last month [73]. The instrument was validated in a Brazilian sample and demonstrated good internal consistency (Cronbach’s α = 0,87) [74].

Mindfulness was assessed using the Portuguese version of the Five Facet Mindfulness Questionnaire (FFMQ), a self-report instrument that evaluates five dimensions: Observing, Acting with Awareness, Nonjudging, Describing, and Nonreacting. The first four dimensions contain eight Likert-scale items each, while the fifth consists of seven. Higher scores indicate greater levels of mindfulness [75]. Each dimension can also be assessed individually, with scores calculated for each subscale. The Portuguese version was validated in Brazil in 2014, demonstrating good internal consistency (Cronbach’s α = 0,81) and high test-retest reliability [76].

### Statistical analysis

Statistical analyses were conducted using IBM Statistical Package for the Social Sciences (SPSS)^®^ version 23 for macOS. A per-protocol analysis was followed, considering only participants who completed at least 50% of the program’s sessions.

In addition to sociodemographic variables, descriptive statistics included the prevalence of depressive and anxiety disorders, calculated as the percentage of participants with scores ≥ 10, based on the clinical cutoffs for the PHQ-9 and GAD-7 (see *Description of measures*). Due to the small sample size and nonnormal distribution of continuous outcome variables, a nonparametric test was chosen. Consequently, paired Wilcoxon signed-rank tests were performed to compare median outcome differences pre- and post-intervention.

The test statistic (*Z*) and its *p*-value were reported alongside the effect size (*r*), calculated using the formula *Z* / √N. Calculating effect sizes is recommended for nonparametric analyses [77] and was interpreted according to Cohen’s criteria: *r* values between 0.1 and 0.3 indicate a small effect, between 0.3 and 0.5 a medium effect, and ≥ 0.5 a large effect [78]. Since SPSS provides *Z* as a standardized statistic, it was reported as positive regardless of the direction of change, which was determined by observing median differences and explicitly described in the results.

A *p*-value < 0.05 was considered statistically significant. All participants completed the questionnaires in full, resulting in no missing data, and analyses were conducted as complete cases.

## Results

Sixteen students were invited to participate, and 13 were assessed for eligibility. All 13 met the inclusion criteria and were enrolled in the MBHP program. Feasibility findings showed that all enrolled participants started the program and attended at least 50% of the sessions, resulting in recruitment and retention rates of 100%.

As part of the safety screening, one participant was invited for a clinical interview with the study psychiatrist and was cleared to participate, as they were receiving ongoing care from a private psychologist and psychiatrist.

Baseline sociodemographic data are presented in Table 1. Participants were mostly female (76.9%) and Caucasian (92.3%), with a mean age of 23.6 years (standard deviation [SD] = 5.23). The majority were in the first two years of medical school, were single, and did not live alone. Based on cutoff scores, the prevalence of major depressive disorder in the sample was 69.2%, while generalized anxiety disorder was 84.6%. Six participants reported using psychiatric medications, including benzodiazepines, selective serotonin reuptake inhibitors, and methylphenidate. Five were under the care of a psychologist and/or psychiatrist, and one participant reported using an herbal remedy (valerian). None engaged in yoga or other formal stress management practices.

**Table 1:** Baseline sociodemographic characteristics (*N* = 13)

Mean Age (SD)	23.6 (5.23)
Gender	
Female	10 (76.9)
Male	3 (23.1)
Living arrangement	
Alone	4 (30.8)
With others	9 (69.2)
Marital Status	
Single	13 (100)
Ethnicity	
Caucasian	12 (92.3)
Mixed race	1 (7.7)
Medical school year	
1st e 2nd	8 (61.5)
3rd e 4th	1 (7.7)
5th e 6th	4 (30.8)
Physical Activity	
Do not practice	8 (61.5)
Practice 1 to 2 h per week	1 (7.7)
Practice 3 to 6 h per week	4 (30.8)
Employment	
No	10 (76.9)
Yes	3(23.1)
Prevalence of Mental Health Disorders	
Major Depression	9 (69.2)
Generalized Anxiety	11 (84.6)

Data is presented as n (%) unless specified otherwise. SD = Standard Deviation

During the program, two participants adjusted their psychiatric medications: one started clonazepam and increased sertraline, while another discontinued clonazepam. One participant began practicing yoga during the program, and a few participants increased or decreased their exercise levels, though no consistent pattern emerged. No new psychological or psychiatric treatments were initiated during the intervention, aside from these reported medication adjustments and lifestyle changes.

The effects of the MBHP program on primary outcomes are presented in Table 2. Median depression scores decreased by 69%, from 13 at baseline to 4 post-intervention (*Z* = 3.18, *p* =.001, *r* =.62). Similarly, anxiety symptoms showed significant reductions, with median scores decreasing by 62.5%, from 16 at baseline to 6 at the eighth week (*Z* = 2.45, *p* =.014, *r* =.41). Perceived stress decreased by 20.5%, from a median score of 39 at baseline to 31 post-intervention, though this reduction was not statistically significant (*Z* = 1.89, *p* =.059, *r* =.37). Finally, mindfulness levels showed a significant 35.8% increase, from a median score of 95 at baseline to 129 at the eighth week (*Z* = 3.18, *p* =.001, *r* =.62).

**Table 2:** Within-group changes in median primary outcome scores from baseline to post-intervention (*N* = 13)

Outcome	Baseline Median	Post-Intervention Median	Z-value	*p*-value	Effect Size (*r*)
*Depression (PHQ-9)*	13	4	**3.18**	**0.001**	0.62
*Anxiety (GAD-7)*	16	6	**2.45**	**0.014**	0.41
*Perceived Stress (PSS-10)*	39	31	1.89	0.059	0.37
*Mindfulness (FFMQ)*	95	129	**3.18**	**0.001**	0.62

PHQ-9, Patient Health Questionnaire-9. GAD-7, Generalized Anxiety Disorder-7. PSS-10, Perceived Stress Scale-10. FFMQ, Five Facet Mindfulness Questionnaire

*Boldface indicates *p* <.05

Table 3 summarizes the effects of the MBHP program on secondary outcomes, which consist of the five mindfulness dimensions assessed individually. “Observing” median scores significantly increased by 31.8%, from 22 at baseline to 29 at the eighth week (*Z* = 2.51, *p* =.012, *r* =.49). “Acting with Awareness” median scores increased significantly by 62.5%, from 16 at baseline to 26 post-intervention (*Z* = 2.62, *p* =.009, *r* =.51). “Nonjudging” median scores demonstrated a significant 35% increase, from 20 at baseline to 27 at the eighth week (*Z* = 1.99, *p* =.046, *r* =.39). Finally, “Nonreacting” mean scores showed a significant 35.7% increase, from 14 at baseline to 19 at the eighth week (*Z* = 2.70, *p* =.007, *r* =.52).

**Table 3:** Within-group changes in median secondary outcome scores from baseline to post-intervention (*N* = 13)

Mindfulness Dimension	Baseline Median	Post-Intervention Median	Z-value	*p*-value	Effect Size (*r*)
*Observing*	22	29	**2.51**	**0.012**	0.49
*Acting with Awareness*	16	26	**2.62**	**0.009**	0.51
*Nonjudging*	20	27	**1.99**	**0.046**	0.39
*Describing*	21	24	1.19	0.233	0.23
*Nonreacting*	14	19	**2.70**	**0.007**	0.52

*Boldface indicates *p* <.05

In contrast, “Describing” median scores did not differ significantly, changing from 21 at baseline to 24 post-intervention (*Z* = 1.19, *p* =.233, *r* =.23).

## Discussion

### Main findings

The findings of this study suggest that the MBHP program is feasible and may help mitigate mental health disorders in medical students. Preliminary results include a significant reduction in depressive symptoms with a large effect size, a significant reduction in anxiety symptoms with a medium effect size, and a significant increase in mindfulness with a large effect size. The effects on perceived stress did not reach statistical significance. However, given the very small sample size (*n* = 13), the study was not powered to adequately test hypotheses; therefore, all results should be interpreted with caution.

Additionally, an exploratory analysis of mindfulness dimensions revealed significant increases in Observing, Nonjudging, Acting with Awareness, and Nonreacting, with the first two demonstrating moderate effect sizes and the latter two showing large effect sizes. The Describing dimension did not change significantly.

### Reductions in depressive symptoms

Depressive symptoms in this study decreased by 69% from pre- to post-intervention, aligning with prior research indicating that MBIs reduce depressive symptoms in medical students [32–34]. Although the systematic review by Daya et al. reports mixed evidence for this outcome, the majority of included trials (67%) that assessed depression showed significant reductions [33]. A recent systematic review and meta-analysis, which included only RCTs, also reported significant reductions in depression immediately following the MBI in three out of four studies (*n* = 307) [32]. However, due to insufficient data, a meta-analysis could not be conducted for this specific outcome.

Nonetheless, both reviews highlight important limitations, including the low quality of evidence in the included studies (measured using validated quality assessments), heterogeneity in intervention protocols and outcome measures, short-term follow-up, and the predominance of small studies with mostly female samples [32, 33]. The need for higher-quality trials in this field remains evident.

Brazilian studies on this topic have been limited [57, 59], with only one assessing depression as an outcome. This study, an RCT with a sample of 141 first-year medical students, did not find a reduction in depression [58]. To our knowledge, the present study is the first to demonstrate a decrease in depressive symptoms following an MBI in Brazilian medical students. The authors of the prior RCT primarily attributed their negative findings to the fact that the course was a required component of the medical school curriculum, unlike most other studies where participation was elective [32–34]. Similarly, other trials involving mandatory MBIs for medical students have also reported negative outcomes [79–81]. Since MBIs are not passive, motivation to engage in both class and home practice is essential. However, the RCT authors noted that some students engaged minimally with the program components, and only a few reported adhering to home practice [58]. Additional factors that may have contributed to the lack of effect include the larger group size in that trial (approximately 45 students per group), which contrasts with the smaller groups typically used in other studies (around 10 to 20 students) [28, 82, 83], including the present study. Finally, the timing of the intervention, which began as early as the second week of medical school, may have been suboptimal, as this period is not typically associated with high stress levels.

Although research on mindfulness and depression in medical students remains limited, findings from other depressive populations are fairly robust. An individual patient data meta-analysis of over 1,200 patients demonstrated the noninferiority of the MBCT program compared to first-line pharmacologic treatment in preventing depression relapse [44].

The high prevalence of depression in both national (30%) and international (28%) samples of medical students [1, 7] underscores the significance of the present study, as MBIs may help mitigate the long-term health and financial consequences for both individual students and the healthcare system [14, 15, 18–21]. Additionally, since higher levels of depression are associated with poor academic performance, increased medical errors, cynicism, and reduced empathy [3], MBIs could potentially enhance medical students’ training. Future studies should assess these outcomes directly.

### Reductions in anxiety symptoms

With respect to anxiety, we observed that the MBHP program reduced symptoms by 62.5% immediately post-intervention. Data on this outcome is somewhat more limited than on depression, with two non-RCTs [84, 85], and one systematic review including four RCTs directly assessing it [32]. Nevertheless, consistent with our findings, the systematic review and meta-analysis by da Silva et al. reported significant reductions in anxiety in three out of four studies involving 307 participants [32]. Similar to depression, the limited data made it unfeasible to conduct a meta-analysis for this outcome.

Findings from the non-RCTs have been mixed: one study reported significant reductions in anxiety in the MBSR group compared to the control group [84], while another, which tested an MBI tailored for medical students developed by Epstein and colleagues [86], found no significant effect [85]. The same limitations discussed previously apply here, particularly the low quality and high risk of bias in trials assessing anxiety [32]. Thus, it is prudent to refrain from drawing definitive conclusions until higher-quality trials are conducted.

The landscape of mindfulness research on anxiety in Brazilian medical students reflects similar challenges. Only the RCT by Damião Neto et al. has assessed this outcome in Brazilian samples, and it did not find significant results, primarily due to the intervention’s compulsory nature [58]. Thus, to our knowledge, this is the first study to demonstrate significant reductions in anxiety among medical students immediately following an MBI.

Despite the mixed evidence in this specific population, the substantial body of research supporting the efficacy of MBIs for anxiety disorders—including a recent RCT of 276 individuals demonstrating the noninferiority of MBSR to first-line pharmacologic treatment [46]—suggests that further trials assessing anxiety in medical students should be encouraged.

### Findings on perceived stress

Regarding perceived stress, our study did not find statistically significant reductions. Previous international and Brazilian research evaluating the effects of MBIs on perceived stress in medical students—as well as related constructs such as psychological distress and subjective stress—has also yielded conflicting results [32–34, 57–59]. Although the meta-analysis by da Silva et al. reported significantly lower stress levels post-intervention and up to a one-year follow-up, the authors rated the risk of bias as moderate, and the effect sizes were small, likely due to substantial heterogeneity among studies [32]. Additionally, a prior review found that only 57% of studies assessing stress reported significant reductions, with one study even documenting an increase in stress [33].

Several factors may explain these discrepant findings. First, interventions that focus solely on the individual aspect of stress may be insufficient, as effective stress reduction may also require institutional-level changes, such as curricular restructuring and pass/fail grading systems [24, 26]. Second, the heterogeneity of stress assessment tools is noteworthy, as different instruments assess distinct aspects of the construct [33]. In our study, we used the PSS-10, which measures subjective perceptions of stress over the past month and does not represent a clinical diagnosis. Moreover, perceived stress scores are known to vary according to age and gender, with higher scores commonly observed among young adults and females [87], characteristics present in our sample. Future studies should prioritize interventions that integrate both the individual and institutional components and may benefit from combining measures that capture only one dimension of stress perception, such as the PSS-10, with complementary assessments— including physiological indicators like salivary cortisol— to provide a more comprehensive understanding of stress responses.

### Increases in mindfulness and its dimensions

Lastly, the present study found that overall mindfulness increased by 35.8%, with four out of five facets — observing, nonjudging, nonreacting, and acting with awareness — also showing significant increases, ranging from 32 to 62%. These findings align with both international and Brazilian literature [32, 58, 59]. Meta-analytic data on 462 medical students suggest that mean mindfulness scores in MBI groups were significantly higher than those in control groups at the end of the intervention. The evidence was rated as high quality, although effect sizes were small [32].

However, Brazilian studies contradict these findings. Both a large RCT and a small single-arm study using the FFMQ reported no significant differences in overall mindfulness scores or any of its five dimensions [58, 59]. To the best of our knowledge, this is the first study to demonstrate significant increases in mindfulness following an MBI in a sample of Brazilian medical students.

Notably, the Describing dimension did not increase, consistent with previous MBHP research [48] and a meta-analysis of 37 RCTs of other MBIs [88]. This suggests that, compared to dimensions such as Acting with Awareness and Non-judging, Describing tends to be less responsive to short-term MBIs. One possible explanation is that MBHP and similar interventions emphasize experiencing sensations and emotions directly, rather than conceptualizing or analyzing them. Although some practices, such as “noting” or “labeling emotions,” aim to help participants name and describe their experiences without becoming caught in analysis, these practices are typically not included in MBHP or other traditional short-term MBIs.

### Limitations

Several limitations must be acknowledged. First, the small sample size (*n* = 13), with a predominantly Caucasian (92%) and female (77%) participant pool, reduced the statistical power and generalizability of our findings. This type of gender selection bias is common in mindfulness research, with one review reporting that at least 73% of participants were female [33]. Although future studies should strive for a more balanced gender distribution, it is important to note that depression and anxiety are more prevalent in women than in men [89].

Second, since participation was voluntary, another form of selection bias may have increased the likelihood of enrolling more motivated participants or those predisposed to respond to MBIs due to personality traits [34]. Conversely, individuals with poorer mental health may have viewed the program as an opportunity to alleviate their distress, which could explain the higher baseline prevalence of generalized anxiety (84.6%) and depression (69.2%) in our sample compared to the national averages of 32.9% and 30.6%, respectively [3–5]. Similarly, other MBI studies on medical students have reported higher baseline levels of mental health distress compared to age-matched samples [90, 91], making this a common occurrence in this field of research. However, this is not a sample of healthy medical students and does not reflect the average prevalence of mental health conditions in this population. Therefore, the generalizability of our findings is limited, and the results should be interpreted with this context in mind.

Although this limits the generalizability of our findings, previous studies on compulsory MBI participation among medical students have largely been unsuccessful [58, 79–81], likely because active engagement in program practices is essential for its efficacy. Notably, students reported significantly higher satisfaction with elective MBSR participation compared to mandatory participation [80]. Hence, despite these limitations, we believe that future studies should continue offering these programs on an optional basis while acknowledging that only a specific subset of medical students may benefit from them.

Third, the single-arm design, absence of a control group, and lack of follow-up assessments beyond the immediate post-intervention period limited the ability to draw causal inferences, rule out placebo effects, and assess whether the effects are long-lasting. Notwithstanding, the per-protocol analysis is a strength of this study, which supports the intervention’s efficacy.

Fourth, this study was retrospectively registered in a clinical trials registry rather than before participant enrollment. Although this does not affect the validity of the findings, prospective registration is recommended to enhance transparency and reduce the risk of selective reporting.

Fifth, some participants were concurrently engaged in mental health and lifestyle treatments, such as psychiatric medications, psychotherapy, and physical activity, which may have contributed to the observed improvements. However, changes in these therapies during the program were minimal and unlikely to fully explain the effects. Nonetheless, we cannot exclude the possibility that these factors influenced the outcomes, and our findings should be interpreted with this limitation in mind.

Sixth, the MBHP curriculum was not particularly tailored to the distinct academic stressors experienced in each year of medical school. While the program focuses on developing transversal coping skills such as emotional regulation, self-awareness, and attention control, these are directed towards individual experiences rather than systematically addressing year-specific challenges. Future adaptations of the MBHP could consider making the program more relevant to the medical school context as a whole, enhancing its applicability across the diverse stages of medical training.

Finally, the impact of the MBI was assessed exclusively through self-report tools, which are inherently prone to response and recall biases. Future studies are encouraged to use an RCT design with longer follow-ups and incorporate physiological markers of stress alongside psychological measures.

## Conclusions

To the best of our knowledge, despite its limitations, this study is the first to demonstrate significant reductions in depressive and anxiety symptoms, as well as increases in mindfulness, following an MBI—specifically the MBHP program—in a sample of Brazilian medical students. Additionally, the program exhibited high feasibility. However, definitive conclusions cannot be drawn until larger RCTs offering elective MBIs to Brazilian medical students are conducted. Pilot trials like this one provide preliminary insights into potential mechanisms and offer effect size estimations to guide future larger trials.

## Data Availability

The datasets used and/or analyzed during the current study are available from the corresponding author on reasonable request.

## Abbreviations

MBHP: Mindfulness–based health promotion
MBI: Mindfulness–based intervention
PHQ: 9–patient health questionnaire–9
GAD: 7–generalized anxiety disorder–7
PSS: 10–perceived stress scale–10
FFMQ: Five facet mindfulness questionnaire
WHO: World health organization
MBSR: Mindfulness–based stress reduction
MBCT: Mindfulness–based cognitive therapy
MB: EAT–mindfulness–based eating awareness training
MBRP: Mindfulness–based relapse prevention
MB: BP–mindfulness–based blood pressure reduction
RCT: Randomized–controlled trial
US: United states
ReBEC: Registro brasileiro de ensaios clínicos
UNIGRANRIO: Universidade do grande rio professor josé de souza herdy
CAAE: Certificado de apresentação para apreciação Ética
UNIFESP: Universidade federal do estado de são paulo
DSM: IV–diagnostic and statistical manual of mental disorders IV
SPSS: Statistical package for the social sciences
SD: Standard deviation

## References

[1] Rotenstein LS, Ramos MA, Torre M, Segal JB, Peluso MJ, Guille C, et al. Prevalence of depression, depressive symptoms, and suicidal ideation among medical students: A systematic review and Meta-Analysis. JAMA. 2016;316(21):2214–36. 27923088 10.1001/jama.2016.17324 PMC5613659

[2] Tian-Ci Quek T, Wai-San Tam W, Tran X, Zhang B, Zhang M, Su-Hui Ho Z. The global prevalence of anxiety among medical students: A Meta-Analysis. Int J Environ Res Public Health. 2019;16(15):2735. 31370266 10.3390/ijerph16152735 PMC6696211

[3] Dyrbye LN, Thomas MR, Shanafelt TD. Systematic review of depression, anxiety, and other indicators of psychological distress among US and Canadian medical students. Acad Med. 2006;81(4):354–73. 16565188 10.1097/00001888-200604000-00009

[4] Suarez DE, Cardozo AC, Ellmer D, Trujillo EM. Cross sectional comparison of anxiety and depression symptoms in medical students and the general population in Colombia. Psychol Health Med. 2021;26(3):375–80. 32314943 10.1080/13548506.2020.1757130

[5] Voltmer E, Kötter T, Spahn C. Perceived medical school stress and the development of behavior and experience patterns in German medical students. Med Teach. 2012;34(10):840–7. 22917267 10.3109/0142159X.2012.706339

[6] de Oliva Costa EF, Santana YS, de Abreu Santos ATR, Martins LAN, de Melo EV, de Andrade TM. Depressive symptoms among medical intern students in a Brazilian public university. Revista Da Associação médica Brasileira. (English Edition). 2012;58(1):53–9. 22392317

[7] Pacheco JP, Giacomin HT, Tam WW, Ribeiro TB, Arab C, Bezerra IM, et al. Mental health problems among medical students in brazil: a systematic review and meta-analysis. Brazilian J Psychiatry. 2017;39:369–78. 10.1590/1516-4446-2017-2223 PMC7111407 28876408

[8] Da Silva VM, Silva-Mendonça V. Symptoms of anxiety and depression and reasons for seeking mental health services among medical students from São Paulo, Brazil. Rev Fac Med. 2024;72(3):e111373.

[9] Dos Santos DT, Nazário FP, Freitas RA, Henriques VM, de Paiva IS. Alcohol abuse and dependence among Brazilian medical students: association to sociodemographic variables, anxiety and depression. J Subst Use. 2019;24(3):285–92.

[10] Hafferty FW. Beyond curriculum reform: confronting medicine’s hidden curriculum. Acad Med. 1998;73(4):403–7. 9580717 10.1097/00001888-199804000-00013

[11] Slavin SJ. Medical student mental health: culture, environment, and the need for change. JAMA. 2016;316(21):2195–6. 27923076 10.1001/jama.2016.16396

[12] MacLean L, Booza J, Balon R. The impact of medical school on student mental health. Acad Psychiatry. 2016;40:89–91. 25749920 10.1007/s40596-015-0301-5

[13] Eley DS, Bansal V, Leung J. Perfectionism as a mediator of psychological distress: implications for addressing underlying vulnerabilities to the mental health of medical students. Med Teach. 2020;42(11):1301–7. 32805157 10.1080/0142159X.2020.1805101

[14] Tyssen R, Vaglum P, Grønvold NT, Ekeberg Ø. Factors in medical school that predict postgraduate mental health problems in need of treatment. A nationwide and longitudinal study. Med Educ. 2001;35(2):110–20. 11169082 10.1046/j.1365-2923.2001.00770.x

[15] World Health Organization. Burn-out an occupational phenomenon: International classification of diseases [Internet]. Geneva: WHO; 2019 May 28 [cited 2025 Jul 24]. https://www.who.int/news/item/28-05-2019-burn-out-an-occupational-phenomenon-international-classification-of-diseases

[16] Maslach C, Jackson SE. Burnout in organizational settings. Appl Soc Psychol Ann. 1984;5:133–53.

[17] Schaufeli WB, Maslach C, Marek T, editors. Professional burnout: Recent developments in theory and research. 1st ed. London: Routledge; 1993. 10.4324/9781315227979

[18] Shanafelt TD, Balch CM, Bechamps G, Russell T, Dyrbye L, Satele D, et al. Burnout and medical errors among American surgeons. Ann Surg. 2010;251(6):995–1000. 19934755 10.1097/SLA.0b013e3181bfdab3

[19] Al-Ghunaim TA, Johnson J, Biyani CS, Alshahrani KM, Dunning A, O’Connor DB. Surgeon burnout, impact on patient safety and professionalism: A systematic review and meta-analysis. Am J Surg. 2022;224(1):228–38. 34974884 10.1016/j.amjsurg.2021.12.027

[20] Panagioti M, Geraghty K, Johnson J, Zhou A, Panagopoulou E, Chew-Graham C, et al. Association between physician burnout and patient safety, professionalism, and patient satisfaction: a systematic review and meta-analysis. JAMA Intern Med. 2018;178(10):1317–31. 30193239 10.1001/jamainternmed.2018.3713 PMC6233757

[21] Han S, Shanafelt TD, Sinsky CA, Awad KM, Dyrbye LN, Fiscus LC, et al. Estimating the attributable cost of physician burnout in the united States. Ann Intern Med. 2019;170(11):784–90. 31132791 10.7326/M18-1422

[22] Strecker EA, Appel KE, Palmer HD, Braceland FJ. Psychiatric studies in medical education: II. Neurotic trends in senior medical students. Am J Psychiatry. 1937;93(5):1197–229.

[23] Shanafelt TD, West CP, Dyrbye LN, Trockel M, Tutty M, Wang H, Carlasare LE, Sinsky C. Changes in burnout and satisfaction with work-life integration in physicians during the first 2 years of the COVID-19 pandemic. InMayo Clinic Proceedings 2022 (Vol. 97, No. 12, pp. 2248–2258). Elsevier. 10.1016/j.mayocp.2022.09.002 PMC9472795 36229269

[24] Wasson LT, Cusmano A, Meli L, Louh I, Falzon L, Hampsey M, et al. Association between learning environment interventions and medical student Well-being: A systematic review. JAMA. 2016;316(21):2237–52. 27923091 10.1001/jama.2016.17573 PMC5240821

[25] Ungar P, Schindler A-K, Polujanski S, Rotthoff T. Online programs to strengthen the mental health of medical students: A systematic review of the literature. Med Educ Online. 2022;27(1):2082909. 35642839 10.1080/10872981.2022.2082909 PMC9176341

[26] Yusoff MSB. Interventions on medical students’ psychological health: a meta-analysis. J Taibah Univ Med Sci. 2014;9(1):1–13.

[27] Shapiro SL, Shapiro DE, Schwartz GER. Stress management in medical education: a review of the literature. Acad Med. 2000;75(7):748–59. 10926029 10.1097/00001888-200007000-00023

[28] Shapiro SL, Schwartz GE, Bonner G. Effects of mindfulness-based stress reduction on medical and premedical students. J Behav Med. 1998;21:581–99. 9891256 10.1023/a:1018700829825

[29] Whitehouse WG, Dinges DF, Orne EC, Keller SE, Bates BL, Bauer NK, et al. Psychosocial and immune effects of self-hypnosis training for stress management throughout the first semester of medical school. Psychosom Med. 1996;58(3):249–63. 8771625 10.1097/00006842-199605000-00009

[30] Jain S, Shapiro SL, Swanick S, Roesch SC, Mills PJ, Bell I, et al. A randomized controlled trial of mindfulness meditation versus relaxation training: effects on distress, positive States of mind, rumination, and distraction. Ann Behav Med. 2007;33:11–21. 17291166 10.1207/s15324796abm3301_2

[31] Shiralkar MT, Harris TB, Eddins-Folensbee FF, Coverdale JH. A systematic review of stress-management programs for medical students. Acad Psychiatry. 2013;37:158–64. 23446664 10.1176/appi.ap.12010003

[32] da Silva CCG, Bolognani CV, Amorim FF, Imoto AM. Effectiveness of training programs based on mindfulness in reducing psychological distress and promoting well-being in medical students: a systematic review and meta-analysis. Syst Reviews. 2023;12(1):79. 10.1186/s13643-023-02244-y PMC10160720 37147732

[33] Daya Z, Hearn JH. Mindfulness interventions in medical education: A systematic review of their impact on medical student stress, depression, fatigue and burnout. Med Teach. 2018;40(2):146–53. 29113526 10.1080/0142159X.2017.1394999

[34] Polle E, Gair J. Mindfulness-based stress reduction for medical students: a narrative review. Can Med Educ J. 2021;12(2):e74–80. 33995723 10.36834/cmej.68406 PMC8105581

[35] Barnes N, Hattan P, Black DS, Schuman-Olivier Z. An examination of Mindfulness-Based programs in US medical schools. Mindfulness. 2017;8(2):489–94.

[36] Kabat-Zinn J, Clinic UMMCWSR. Full catastrophe living: using the wisdom of your body and Mind to face stress. pain, and illness: Delta; 1990.

[37] Sipe WE, Eisendrath SJ. Mindfulness-based cognitive therapy: theory and practice. Can J Psychiatry. 2012;57(2):63–9. 22340145 10.1177/070674371205700202

[38] Lattimore P. Mindfulness-based emotional eating awareness training: taking the emotional out of eating. Eating and weight Disorders-Studies on anorexia. Bulimia Obes. 2020;25(3):649–57. 10.1007/s40519-019-00667-y PMC7256094 30859465

[39] Grant S, Colaiaco B, Motala A, Shanman R, Booth M, Sorbero M, et al. Mindfulness-based relapse prevention for substance use disorders: A systematic review and meta-analysis. J Addict Med. 2017;11(5):386–96. 28727663 10.1097/ADM.0000000000000338 PMC5636047

[40] Lima LC, Mendes LC. Mindfulness and psychological well-being: effects of a mindfulness-based health promotion program on healthy adults. Trends Psychol. 2020;28(2):213–29.

[41] Loucks EB, Neves VV, Cafferky V, Scarpaci MM, Kronish IM. Sustainability of blood pressure reduction through adapted mindfulness training: the MB-BP study. Am J Cardiol. 2024;217:31–4. 38447891 10.1016/j.amjcard.2024.02.012 PMC11067945

[42] Loucks EB, Nardi WR, Gutman R, Kronish IM, Saadeh FB, Li Y, et al. Mindfulness-based blood pressure reduction (MB-BP): stage 1 single-arm clinical trial. PLoS ONE. 2019;14(11):e0223095. 31774807 10.1371/journal.pone.0223095 PMC6881004

[43] Loucks EB, Schuman-Olivier Z, Saadeh FB, Scarpaci MM, Nardi WR, Proulx JA, et al. Effect of adapted mindfulness training in participants with elevated office blood pressure: the MB‐BP study: A randomized clinical trial. J Am Heart Association. 2023;12(11):e028712. 10.1161/JAHA.122.028712 PMC10381983 37218591

[44] Kuyken W, Warren FC, Taylor RS, Whalley B, Crane C, Bondolfi G, et al. Efficacy of mindfulness-based cognitive therapy in prevention of depressive relapse: an individual patient data meta-analysis from randomized trials. JAMA Psychiatry. 2016;73(6):565–74. 27119968 10.1001/jamapsychiatry.2016.0076 PMC6640038

[45] National Institute for Health and Care Excellence (NICE). Depression in adults: treatment and management. London: NICE; 2022. (NICE Guideline No. 222). 35977056

[46] Hoge EA, Bui E, Mete M, Dutton MA, Baker AW, Simon NM. Mindfulness-Based stress reduction vs Escitalopram for the treatment of adults with anxiety disorders: A randomized clinical trial. JAMA Psychiatry. 2023;80(1):13–21. 36350591 10.1001/jamapsychiatry.2022.3679 PMC9647561

[47] Mapurunga MV, Rodrigues de Oliveira D, Andreoni S, Sarubbi V Jr, Bonilha AC, D’Almeida V, Tomita L, Ramos LR, Demarzo M. Effects of mindfulness training on quality of life and well‑being in older adults: A randomized controlled trial. Aging Ment Health. 2025;[Epub ahead of print]:1–13. 10.1080/13607863.2025.2488890 10.1080/13607863.2025.2488890 40220313

[48] Quiroz-González E, Lupano Perugini ML, Delgado-Abella LE, Arenas-Granada J, Demarzo M. Effects of a mindfulness-based health promotion program on mindfulness, psychological capital, compassion fatigue, and affect in healthcare workers. Front Psychol. 2024;15:1470695. 39545141 10.3389/fpsyg.2024.1470695 PMC11561754

[49] Wilson D, de Oliveira DR, Palace-Berl F, de Mello Ponteciano B, Rissatti LF, Pollizi VP, et al. Fostering emotional self-regulation in female teachers at the public teaching network: A mindfulness-based intervention improving psychological measures and inflammatory biomarkers. Brain Behav immunity-health. 2022;21:100427. 10.1016/j.bbih.2022.100427 PMC8881415 35243406

[50] de Oliveira DR, Wilson D, Palace-Berl F, de Mello Ponteciano B, Rissatti LF, de Miranda FS, et al. Mindfulness meditation training effects on quality of life, immune function and glutathione metabolism in service healthy female teachers: A randomized pilot clinical trial. Brain Behav immunity-health. 2021;18:100372. 10.1016/j.bbih.2021.100372 PMC8566766 34761243

[51] Trombka M, Demarzo M, Campos D, Antonio SB, Cicuto K, Walcher AL, et al. Mindfulness training improves quality of life and reduces depression and anxiety symptoms among Police officers: results from the POLICE study—a multicenter randomized controlled trial. Front Psychiatry. 2021;12:624876. 33716824 10.3389/fpsyt.2021.624876 PMC7952984

[52] Oliveira L, Kim CA, Palomo P, Melo DG, Sarubbi Jr V, Oliveira D et al. Mindfulness-based intervention improves quality of life and reduces burden in family caregivers of people with intellectual disabilities: A pragmatic randomized trial with an active control. Qeios. 2025. 10.32388/Q5GB40

[53] Wießner I, Souza JP, Demarzo M, Tófoli LF. Mindfulness enhancements predict aberrant salience reductions and improve stress management. Discover Mental Health. 2025;5(1):1–17. 40199816 10.1007/s44192-025-00179-5 PMC11979052

[54] Santos de Jesus M, Winckler de Oliveira Filho C, Gebara L, Solé Cases S, Maria Cunha Bitencourt Santos S, Sarubbi Jr V, et al. Acceptability, feasibility, and effects of a mindfulness-based intervention on the quality of life and performance of the Brazilian Paralympic Boccia Team: Mixed methods case study. Qeios. 2025. 10.32388/PYEDEK.2

[55] Teixeira DS, Fortes S, Kestenberg C, Alves K, Campos MR, Neto AO, et al. Improving patient-centered mental health promotion in primary care in vulnerable communities through mindfulness training in Rio de janeiro, Brazil. Front Med. 2024;11:1356040. 10.3389/fmed.2024.1356040 PMC11261806 39040898

[56] Salvo V, Curado DF, Sanudo A, Kristeller J, Schveitzer MC, Favarato ML, et al. Comparative effectiveness of mindfulness and mindful eating programmes among low-income overweight women in primary health care: A randomised controlled pragmatic study with psychological, biochemical, and anthropometric outcomes. Appetite. 2022;177:106131. 35753441 10.1016/j.appet.2022.106131

[57] Araujo ACd S, CLAd, Kozasa EH, Lacerda SS, Tanaka LH. Effects of a mindfulness meditation course on healthcare students in Brazil. Acta Paulista De Enfermagem. 2020;33:eAPE20190170.

[58] Damião Neto A, Lucchetti ALG, da Silva Ezequiel O, Lucchetti G. Effects of a required large-group mindfulness meditation course on first-year medical students’ mental health and quality of life: a randomized controlled trial. J Gen Intern Med. 2020;35:672–8. 31452038 10.1007/s11606-019-05284-0 PMC7080902

[59] Carvalho Filho CV, Matos MTL, de Andrade MM, de Souza NCVF, Valadão AF, Cunha ÂGJ, et al. Case series: evaluation of a Mindfulness-based intervention in perceived stress and quality of life of medical students. Brazilian J Health Rev. 2022;5(5):20155–73. Série de casos: avaliação de uma intervenção baseada em Mindfulness no estresse percebido e qualidade de vida de estudantes de medicina.

[60] Demarzo MMP, Andreoni S, Sanches N, Perez S, Fortes S, Garcia-Campayo J. Mindfulness-based stress reduction (MBSR) in perceived stress and quality of life: an open, uncontrolled study in a Brazilian healthy sample. Explore: J Sci Healing. 2014;10(2):118–20. 10.1016/j.explore.2013.12.005 24607079

[61] Lancaster GA, Dodd S, Williamson PR. Design and analysis of pilot studies: recommendations for good practice. J Eval Clin Pract. 2004;10(2):307–12. 15189396 10.1111/j..2002.384.doc.x

[62] Thabane L, Ma J, Chu R, Cheng J, Ismaila A, Rios LP, et al. A tutorial on pilot studies: the what, why and how. BMC Med Res Methodol. 2010;10(1):1. 20053272 10.1186/1471-2288-10-1 PMC2824145

[63] Eldridge SM, Chan CL, Campbell MJ, Bond CM, Hopewell S, Thabane L, et al. CONSORT 2010 statement: extension to randomised pilot and feasibility trials. BMJ. 2016;355:i5239. 27777223 10.1136/bmj.i5239 PMC5076380

[64] Billingham SAM, Whitehead AL, Julious SA. An audit of sample sizes for pilot and feasibility trials being undertaken in the united Kingdom registered in the united Kingdom clinical research network database. BMC Med Res Methodol. 2013;13(1):104. 23961782 10.1186/1471-2288-13-104 PMC3765378

[65] Demarzo M, Montero-Marin J, Puebla-Guedea M, Navarro-Gil M, Herrera-Mercadal P, Moreno-González S et al. Efficacy of 8- and 4-Session Mindfulness-Based interventions in a Non-clinical population: A controlled study. Front Psychol. 2017;8. 10.3389/fpsyg.2017.01343 PMC5550824 28848465

[66] Cebolla A, Demarzo M, Martins P, Soler J, Garcia-Campayo J. Unwanted effects: is there a negative side of meditation? A multicentre survey. PLoS ONE. 2017;12(9):e0183137. 28873417 10.1371/journal.pone.0183137 PMC5584749

[67] Demarzo M. Mindfulness y Promoción de la Salud. Revista inspira. 2020;2:20–5.

[68] Kroenke K, Spitzer RL, Williams JB. The PHQ-9: validity of a brief depression severity measure. J Gen Intern Med. 2001;16(9):606–13. 11556941 10.1046/j.1525-1497.2001.016009606.x PMC1495268

[69] de Lima Osório F, Vilela Mendes A, Crippa JA, Loureiro SR. Study of the discriminative validity of the PHQ-9 and PHQ‐2 in a sample of Brazilian women in the context of primary health care. Perspect Psychiatr Care. 2009;45(3):216–27. 19566694 10.1111/j.1744-6163.2009.00224.x

[70] Yoon S, Lee Y, Han C, Pae C-U, Yoon H-K, Patkar AA, et al. Usefulness of the patient health Questionnaire-9 for Korean medical students. Acad Psychiatry. 2014;38:661–7. 24804631 10.1007/s40596-014-0140-9

[71] Spitzer RL, Kroenke K, Williams JB, Löwe B. A brief measure for assessing generalized anxiety disorder: the GAD-7. Arch Intern Med. 2006;166(10):1092–7. 16717171 10.1001/archinte.166.10.1092

[72] Moreno AL, DeSousa DA, Souza AMFLPd, Manfro GG, Salum GA, Koller SH, et al. Estructura factorial, confiabilidad, y ítems parámetros de La versión de Portugués Brasileño Del cuestionario GAD-7. Temas Em Psicologia. 2016;24(1):367–76.

[73] Cohen S, Kamarck T, Mermelstein R. A global measure of perceived stress. J Health Soc Behav. 1983;24(4):385–96. 6668417

[74] Siqueira Reis R, Ferreira Hino AA. Romélio Rodriguez Añez C. Perceived stress scale: reliability and validity study in Brazil. J Health Psychol. 2010;15(1):107–14. 20064889 10.1177/1359105309346343

[75] Baer RA, Smith GT, Hopkins J, Krietemeyer J, Toney L. Using self-report assessment methods to explore facets of mindfulness. Assessment. 2006;13(1):27–45. 16443717 10.1177/1073191105283504

[76] Barros VVd, Kozasa EH, Souza ICWd, Ronzani TM. Validity evidence of the Brazilian version of the five facet mindfulness questionnaire (FFMQ). Psicologia: Teoria E Pesquisa. 2014;30:317–27.

[77] Pallant J. SPSS survival manual: A step by step guide to data analysis using. IBM SPSS: Taylor & Francis 2020.

[78] Cohen J. Statistical power analysis for the behavioral sciences. routledge. 2013.

[79] Dyrbye LN, Shanafelt TD, Werner L, Sood A, Satele D, Wolanskyj AP. The impact of a required longitudinal stress management and resilience training course for first-year medical students. J Gen Intern Med. 2017;32:1309–14. 28861707 10.1007/s11606-017-4171-2 PMC5698225

[80] Aherne D, Farrant K, Hickey L, Hickey E, McGrath L, McGrath D. Mindfulness based stress reduction for medical students: optimising student satisfaction and engagement. BMC Med Educ. 2016;16:1–11. 27535243 10.1186/s12909-016-0728-8 PMC4989331

[81] Hassed C, De Lisle S, Sullivan G, Pier C. Enhancing the health of medical students: outcomes of an integrated mindfulness and lifestyle program. Adv Health Sci Educ. 2009;14:387–98. 10.1007/s10459-008-9125-3 18516694

[82] Phang CK, Mukhtar F, Ibrahim N, Keng S-L, Mohd. Sidik S. Effects of a brief mindfulness-based intervention program for stress management among medical students: the Mindful-Gym randomized controlled study. Adv Health Sci Educ. 2015;20:1115–34. 10.1007/s10459-015-9591-3 25697124

[83] Greeson JM, Toohey MJ, Pearce MJ. An adapted, four-week mind–body skills group for medical students: reducing stress, increasing mindfulness, and enhancing self-care. Explore. 2015;11(3):186–92. 25792145 10.1016/j.explore.2015.02.003

[84] Rosenzweig S, Reibel DK, Greeson JM, Brainard GC, Hojat M. Mindfulness-based stress reduction lowers psychological distress in medical students. Teach Learn Med. 2003;15(2):88–92. 12708065 10.1207/S15328015TLM1502_03

[85] Danilewitz M, Bradwejn J, Koszycki D. A pilot feasibility study of a peer-led mindfulness program for medical students. Can Med Educ J. 2016;7(1):e31. PMC4830371 27103950

[86] Epstein RM, Mindful Practice. JAMA. 1999;282(9):833–9. 10478689 10.1001/jama.282.9.833

[87] Cohen S, Janicki-Deverts D. Who’s stressed? Distributions of psychological stress in the united States in probability samples from 1983, 2006, and 2009 1. J Appl Soc Psychol. 2012;42(6):1320–34.

[88] Baer R, Gu J, Cavanagh K, Strauss C. Differential sensitivity of mindfulness questionnaires to change with treatment: A systematic review and meta-analysis. Psychol Assess. 2019;31(10):1247–63. 31368738 10.1037/pas0000744 PMC6793937

[89] Albert PR. Why is depression more prevalent in women? J Psychiatry Neurosci. 2015;40(4):219–21. 10.1503/jpn.150205 PMC4478054 26107348

[90] van Dijk I, Lucassen PLBJ, Speckens AEM. Mindfulness training for medical students in their clinical clerkships: two cross-sectional studies exploring interest and participation. BMC Med Educ. 2015;15(1):24. 25888726 10.1186/s12909-015-0302-9 PMC4348100

[91] Warnecke E, Quinn S, Ogden K, Towle N, Nelson MR. A randomised controlled trial of the effects of mindfulness practice on medical student stress levels. Med Educ. 2011;45(4):381–8. 21401686 10.1111/j.1365-2923.2010.03877.x

